# Relevant topics in Geriatric Forensic Psychiatry

**DOI:** 10.1192/j.eurpsy.2022.120

**Published:** 2022-09-01

**Authors:** K. Goethals

**Affiliations:** Antwerp University Hospital, University Forensic Centre, Edegem, Belgium

**Keywords:** disinhibition, criminal behaviour, old age, forensic

## Abstract

Aging persons can become involved in the criminal justice system, more commonly as victims but also as offenders. They are a growing group of interest in forensic psychiatry, due to the ageing of the population. Moreover, they are overrepresented in long-stay facilities. Forensic psychiatrists may be asked to evaluate elderly individuals whose behaviour has become problematic to their families, caregivers, or third parties. We will focus here on problematic behaviors in eldery people, particularly disinhibition, agitation and aggression, and criminal behaviour and the incarcerated eldery. Forensic psychiatric assessment with new-onset criminal behaviour require special inquiries regarding criminal responsibility or competency to stand trial. Little research is available regarding criminal behaviour in eldery persons in correctional settings. In this paper a forensic-psychiatric expert report will illustrate these topics.

**Disclosure:**

No significant relationships.

